# Benzo[a]pyrene Exposure Reduces Cell-Type Diversity and Stimulates Sex-Biased Damage Pathways in End Organs of Lupus-Prone Mice

**DOI:** 10.3390/ijms24076163

**Published:** 2023-03-24

**Authors:** Runqi Zhu, Kameron Kennicott, Yun Liang

**Affiliations:** 1 Department of Physiology, Michigan State University, East Lansing, MI 48823, USA; 2 Department of Pharmacology and Toxicology, Michigan State University, East Lansing, MI 48823, USA

**Keywords:** autoimmune diseases, environmental exposure, sex differences, molecular mechanism

## Abstract

Studies indicate that genetic factors only account for approximately thirty percent of all autoimmune diseases, while the rest of autoimmune pathogenesis is attributed to environmental factors including toxic chemicals. To understand if and how environmental pollutants trigger autoimmunity, we investigated the effect of benzo[a]pyrene (BaP) exposure on the development of autoimmune phenotypes in the lupus-prone MRL strain. The exposure of MRL mice to BaP over the course of 8 weeks before lupus onset resulted in total body weight loss in males, while marginal changes in anti-dsDNA levels occurred. Multi-organ analyses of BaP-treated and control MRL mice suggested that the kidney is a major organ directly affected by the metabolism of benzene-containing compounds, with increased expression of BaP-target genes including *Cyp4b1* and *Hao2*. Intriguingly, spatial transcriptomic data showed that BaP caused a drastic reduction in cell-type diversity in both the kidneys and spleen of MRL mice. Further analysis of the molecular pathways affected suggested a sex-biased effect of BaP treatment, with the upregulated expression of angiogenesis genes in the lungs and an increased deposition of C3 in the kidneys of male mice. While SLE is more common in women, the disease is more severe in male patients, with an increased risk of disease progression to renal failure and lung cancer. Our results reveal sex-biased molecular pathways stimulated by BaP which may help explain the increased likelihood of end organ damage in males with lupus.

## 1. Introduction

Autoimmune diseases, commonly characterized by the loss of tolerance and the attack of self-organs by one’s immune system, have multifactorial etiologies [1]. While predisposing genetic risk factors have been identified by the analysis of rare Mendelian autoimmunity syndromes and genome-wide association studies (GWAS), it is estimated that they only account for approximately thirty percent of autoimmune disease pathogenesis [2,3,4]. The remaining seventy percent of risk factors are attributed to environmental pollutants, dietary components, and infections [1,2,3,4]. Therefore, it is important to define how environmental factors contribute to autoimmune disease pathogenesis in order to ultimately develop effective clinical management strategies for these diseases.

A number of environmental toxicants have been associated with autoimmune diseases. For example, mercury (Hg) exposure leads to an increased production of pro-inflammatory cytokines, and silica exposure results in elevated autoantibodies and anti-topoisomerase I and anti-Fas antibodies [5,6,7]. In this study, we focused on benzo(a)pyrene (BaP) because of its widespread presence, with over 70% of the European population exposed to concentrations above the WHO guideline value, according to the Air Quality Report by the European Environment Agency, for example [8]. As one of the most extensively studied environmental pollutants, BaP is a polycyclic aromatic hydrocarbon (PAH) commonly found in byproducts of industrial incineration, motor vehicle emissions, and cigarette smoke [9,10]. BaP has been shown to suppress the production of IgM and IgG in an in vitro antibody response model of splenic suspensions, and the exposure of B6C3F1 mice to BaP resulted in a reduced number of IgM and IgG antibody plaque-forming cells in response to antigens including sheep red blood cells (SRBC) and lipopolysaccharide (LPS) [11,12]. The administration of BaP to Riv:Tox Wistar specific pathogen-free rats resulted in a decreased number of lymphocytes and eosinophilic granulocytes at a high dose (90 mg/kg) [13]. Studies of the effect of BaP on autoimmune-predisposed animals are limited, but it has been shown that a range of BaP doses (5, 20, 40 mg/kg) did not increase anti-dsDNA antibody levels in five-week-old female NZBW/F1 mice [14]. However, a high dose of BaP (40 mg/kg) caused splenic red pulp expansion and a decrease in splenic total B cells and T cells [14].

Consistent with these studies, immunosuppression was indicated to be the most common effect after exposure to PAH in general, while evidence also exists indicating that low doses of PAH can be immunostimulatory [10,13,14,15]. Indeed, levels of nine monohydroxylated urinary PAH metabolites as well as the interaction between PAH exposure and smoking have been found to positively correlate with the prevalence of late-stage rheumatoid arthritis [16]. Exposure to 2-aminoanthracene (2AA), a PAH, was shown to increase the risk for type 1 diabetes [17]. PM_10_ concentration was linked to multiple sclerosis severity in both human and animal studies [18,19]. In addition, the rate of systemic lupus erythematosus (SLE) onset exhibited a positive association with the smoking status and exposure to air pollutants from population studies [20,21]. Studies have also identified an association between smoking and a higher SLEDAI (Systemic Lupus Erythematosus Disease Activity Index) and the progression of lupus nephritis to end-stage renal disease [22,23]. Although these lines of evidence indicate that PAH can accelerate autoimmune pathogenesis under certain scenarios, the exact molecular pathways affected by PAH exposure in autoimmune diseases remain unclear.

To better understand the impact of environmental factors on autoimmune pathogenesis, we sought to elucidate the molecular consequences of BaP exposure using the spontaneous lupus model MRL mouse as a defined experimental system. Despite carrying the normal *Fas* gene, MRL mice exhibit multiorgan, lupus-like disorders including glomerulonephritis, arthritis, skin rash, cerebritis, and anti-dsDNA at a late stage (18 months) [24,25]. The anti-dsDNA antibodies are frequently present in SLE patients (at least 70%) and are historically considered to be sensitive and specific (57.3% and 97.4%, respectively) as diagnostic criteria of SLE [26,27]. Studies have shown that anti-dsDNA antibodies can be detected in 55 percent of SLE patients years before disease diagnosis and that anti-dsDNA positivity is correlated with renal involvement [28,29]. However, it is unclear whether anti-dsDNA antibodies are genuinely part of SLE pathogenesis, and these antibodies can also be detected in patients with autoimmune hepatitis (up to 30%) [30,31]. In addition, like many other autoimmune diseases, SLE is strongly sex-biased, affecting women nine times more frequently than men, a feature recapitulated by the MRL model [24,25,32,33,34]. Consistent with previous studies, we found that chronic exposure to BaP (5 mg/kg) before lupus onset did not significantly elevate anti-dsDNA levels. However, BaP caused a drastic reduction in cell-type diversity in the spleen and kidney of MRL mice from spatial transcriptomic experiments. Further analysis of the molecular pathways affected suggested a sex-biased effect of BaP treatment, with upregulated expression of angiogenesis genes in the lungs and the increased deposition of C3 in the kidneys of male mice. Intriguingly, while SLE is more common in women, the disease is more severe in male patients, and it is known that the male sex is a significant risk factor for disease progression to renal failure and lung cancer, especially in smokers [32,35,36,37]. Our results reveal sex-biased molecular pathways stimulated by BaP, which may help explain the increased likelihood of end organ damage in males with lupus.

## 2. Results

### 2.1. BaP Exposure Caused Immunosuppression in Female MRL Mice

**Figure 1:**
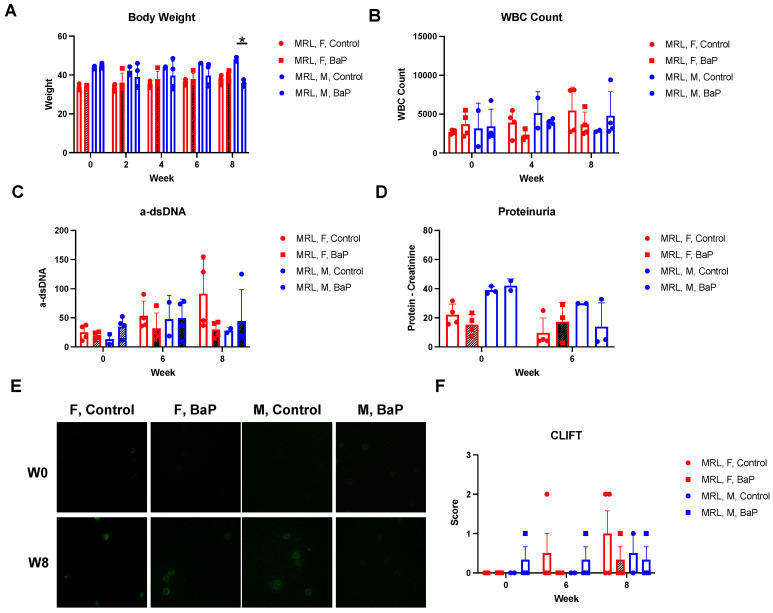
BaP exposure reduced the body weight of male MRL mice. Body weight (**A**), white blood cell count (**B**), a-dsDNA titer (**C**), proteinuria (**D**), and CLIFT assay ((**E**), immunofluorescence (10×) and (**F**), scoring) in female (F) or male (M) MRL mice at the given time-point post-BaP treatment. Control, vehicle-treated. BaP, BaP-treated. W0, week 0. W8, week 8. *, *p* < 0.05.

To understand the effect of BaP exposure on autoimmune-predisposed animals, we subjected MRL mice to BaP treatment over the course of 8 weeks, starting at 8 weeks of age, before lupus onset. To model the common absorption of air BaP through the lungs and skin in humans, the lungs and skin of mice were exposed to BaP, following established procedures: a total dose of 5 mg/kg, which is well within the range of previous studies on the effect of BaP on immune responses in rodents [13,14,38,39]. We found that BaP exposure did not significantly affect the weight of female mice, but male mice experienced ~26% weight loss at 8 weeks of treatment (Figure 1A). There was no significant elevation of white blood cell count, a-dsDNA, or proteinuria to pathological levels over the course of treatment (Figure 1B–D). a-dsDNA measurement was performed using both ELISA and *Crithida luciliae* assays, which showed that while a positive signal was observed in some animals, there were no statistically significant changes after quantification (Figure 1C,E,F). These results are consistent with the fact that MRL mice develop lupus-like phenotypes late in life, using laboratory measurements of antinuclear antibody, glomerulonephritis, vasculitis, and skin lesion development, as well as previous reports indicating that, at this dose, BaP treatment did not significantly alter the levels of white blood cell count, serum a-dsDNA, or proteinuria [14,24,25].

**Figure 2:**
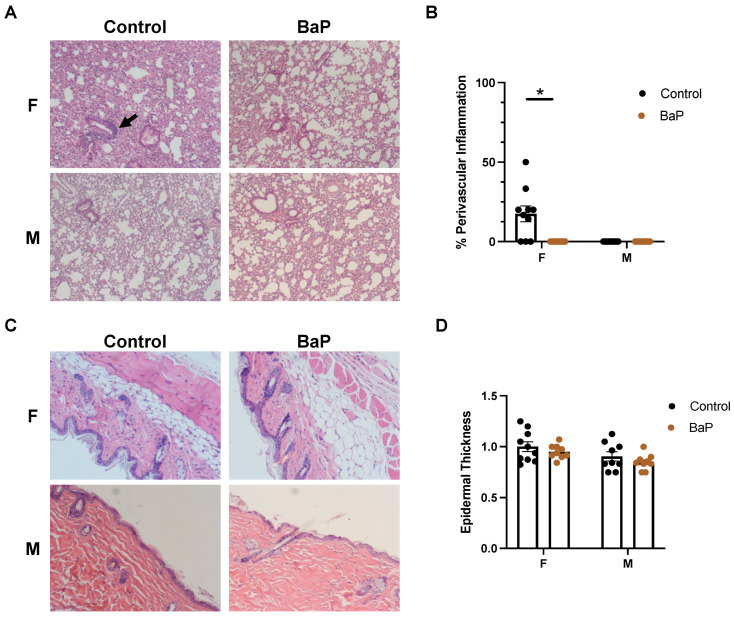
BaP exposure reduced perivascular inflammation in the lung of female MRL mice. (**A**) H&E staining of female (F) or male (M) MRL lung upon vehicle (control) or BaP (BaP) treatment, with the arrow pointing to the site of perivascular inflammation (4×). (**B**) Quantification of perivascular inflammation of bronchioles in female (F) or male (M) vehicle (control) or BaP (BaP)-treated lungs. *, *p* < 0.05, Student’s *t*-test. (**C**) H&E staining of female (F) or male (M) MRL skin upon vehicle (control) or BaP (BaP) treatment (4×). (**D**) Quantification of epidermal thickness in female (F) or male (M) vehicle (control) or BaP (BaP)-treated skin.

Intriguingly, H&E staining suggested the perivascular inflammation of bronchioles in the lungs of female MRL mice (Figure 2A,B). On the contrary, there were no significant bronchiole findings in male MRL mice (Figure 2A,B), which is consistent with the known female bias of the model [24,25]. There were no significant alveoli findings in either female or male mice. BaP treatment reduced the level of perivascular inflammation in the lungs of the female mice, while causing no detectable changes in males (Figure 2A,B). No skin rash developed throughout the course of the treatment, and correspondingly, there were no significant pathological findings in the skin of female or male MRL mice, regardless of BaP treatment (Figure 2C,D).

**Figure 3:**
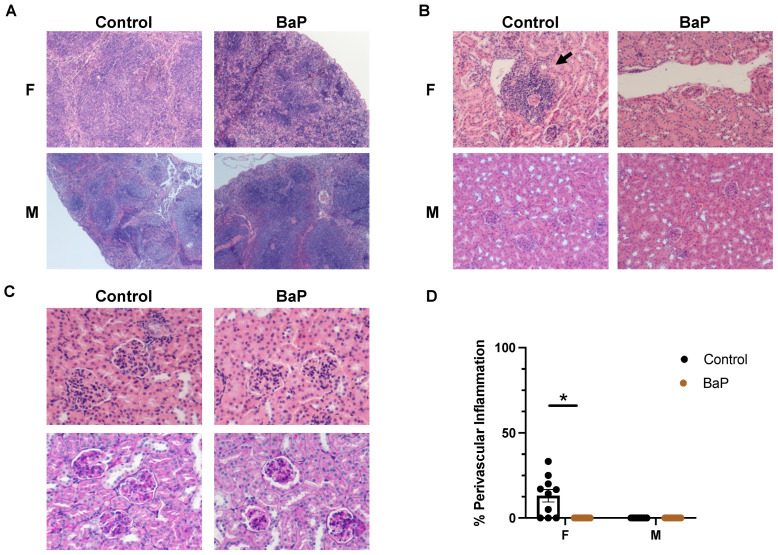
BaP exposure disrupted white pulp in the spleen and reduced inflammation in the kidney of female MRL mice. (**A**) H&E staining of female (F) or male (M) MRL spleen upon vehicle (control) or BaP (BaP) treatment (4×). (**B**) H&E staining of female (F) or male (M) MRL kidney upon vehicle (control) or BaP (BaP) treatment, with the arrow pointing to the site of perivascular inflammation in the cortex (4×). (**C**) H&E (top) and PAS (below) staining of glomeruli in female MRL mice upon vehicle (control) or BaP (BaP) treatment (4×). (**D**) Quantification of arteriolar inflammation in the renal cortex of female (F) or male (M) vehicle (control) or BaP (BaP)-treated kidney. *, *p* < 0.05, Student’s *t*-test.

Internally, BaP treatment caused a drastic disruption of the white pulp in the spleen of female MRL mice, with a concomitant increase in the red pulp (Figure 3A). In contrast, no disorganization in the white pulp was observed in male MRL mice upon treatment (Figure 3A).

Similar to the lungs, we detected arteriolar inflammation in the renal cortex of female MRL mice, which was decreased after BaP treatment, while there were no significant findings in the kidneys of male MRL mice (Figure 3B,D). Despite the observed inflammation, renal pathology has not progressed to the stage with the thickening of the glomerular basement membrane, as shown by H&E and PAS staining (Figure 3C).

Collectively, we confirmed that our model depicted the ‘pre-disease onset’ stage in lupus pathogenesis, before a-dsDNA and proteinuria reached pathological levels. However, there was already evidence of inflammation in the lungs and kidneys of female MRL mice. BaP exposure caused immunosuppression, including the reduction in lymphocyte infiltration and the disruption of the white pulp in female MRL mice. While no significant pathological findings were evident from H&E staining, male MRL mice experienced a loss of body weight, a sign of sickness not seen in females.

### 2.2. BaP Treatment Stimulated the Expression of Vasculature Genes in the Lungs of MRL Mice

**Figure 4:**
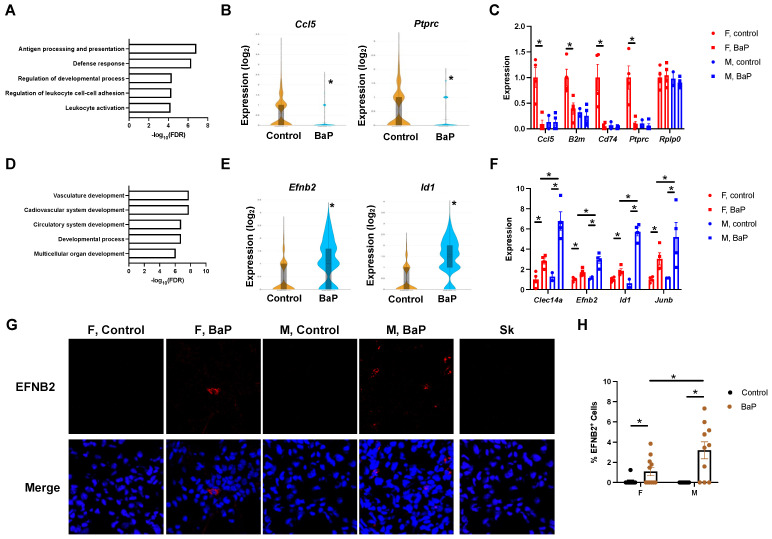
BaP treatment stimulated the expression of vasculature genes in the MRL lung in a male-biased manner. (**A**) Top gene ontology categories enriched in genes downregulated by BaP treatment in female MRL lungs, with example genes shown in (**B**). (**C**) Validation of BaP-downregulated, immune-associated genes by qPCR. (**D**) Top gene ontology categories enriched in genes upregulated by BaP treatment in female MRL lungs, with example genes shown in (**E**). (**F**) Validation of BaP-upregulated, pro-angiogenesis genes by qPCR. (**G**) Immunostaining of EFNB2 in female or male control- or BaP-treated lungs, with staining in skin as a negative control and quantification in (**H**) (20×). F, female. M, male. Control, vehicle-treated. BaP, BaP-treated. Sk, skin. * *p* < 0.05, Student’s *t*-test.

Because we detected the immunosuppressive effect of BaP treatment on female MRL mice, we performed spatial transcriptomic studies on the female MRL lung, skin, spleen, and kidney, comparing untreated vs. BaP-treated conditions. Differential gene expression analysis of untreated vs. BaP-treated female MRL lungs identified 19 downregulated genes and 37 upregulated genes at FDR < 0.05 (Supplementary Figures S1 and S2). Gene ontology analysis suggested that BaP-downregulated genes were enriched in biological functions including antigen processing and presentation, defense response, and leukocyte activation, which is consistent with the previous observation that BaP reduced lung inflammation in female MRL mice (Figure 4A, Supplementary Figure S2B). Immune-associated genes downregulated by BaP included *Ccl5*, which is also called RANTES (Regulated upon Activation, Normal T Cell Expressed, and Presumably Secreted) and is known to mediate eosinophil, neutrophil, and monocyte recruitment to the airways and initiate important events in the inflammatory response [40,41], and *Ptprc*, also known as the CD45 antigen, which is a central regulator of hematopoietic cell differentiation and autoimmunity [42] (Figure 4B,C).

Intriguingly, BaP-upregulated genes were enriched in functional categories including vasculature and circulatory system development (Figure 4D, Supplementary Figure S4C). These vasculature genes included *Efnb2*, a member of the ephrin family and a pro-angiogenic factor whose overexpression predicts the poor prognosis of solid tumors [43,44,45], and *Id1*, a helix–loop–helix transcription factor that regulates tumor angiogenesis [46] (Figure 4E,F). BaP upregulated the expression levels of these pro-angiogenic and pro-tumorigenic factors [47,48,49] in male MRL lungs more than in their female counterparts (Figure 4F). Immunofluorescence staining also showed that there was an increase in the number of EFNB2-positve cells in the lungs of BaP-treated MRL mice, an effect more substantial in males than females (Figure 4G,H). The BaP treatment of human cultured endothelial cells (HUVECs) in vitro upregulated a subset of the BaP targets in MRL lungs, including *EFNB2* and *ID1*, suggesting that pro-autoimmune processes in vivo facilitate the full activation of BaP targets (Supplementary Figure S3).

Similar to the lack of significant findings in skin pathology, spatial transcriptomic analysis of BaP-treated vs. non-treated MRL mouse skin did not return molecular differences (Supplementary Figure S4).

Therefore, the molecular analysis of MRL organs directly exposed to BaP confirmed the immunosuppressive effect of BaP in females and identified angiogenesis genes as primary targets upregulated by BaP in the lung. The stimulatory effects of BaP on angiogenic and tumorigenic genes including *Clec14a*, *Efnb2*, *Id1*, and *Junb* were higher in males than in females, which is consistent with the finding that the male sex is a significant risk factor for lung cancer in SLE patients, especially among smokers [35,37].

### 2.3. BaP Treatment Disrupted the T Cell Compartment in the Spleen of Female MRL Mice

**Figure 5:**
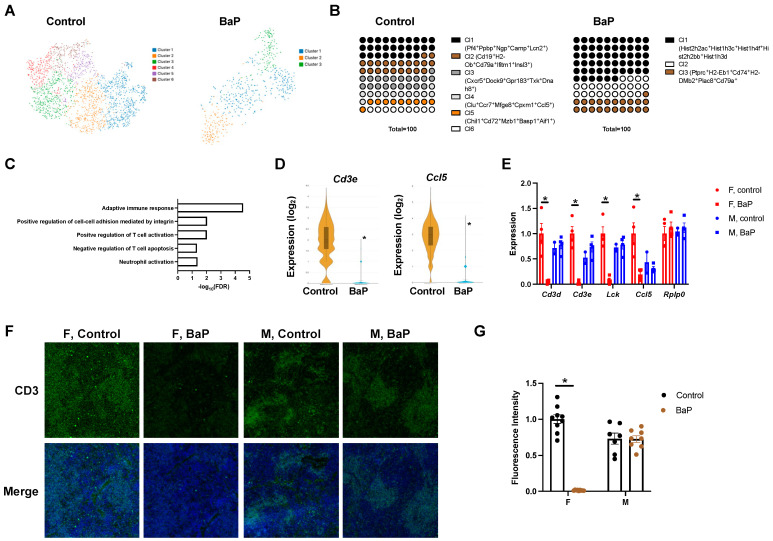
BaP treatment reduced cell-type diversity in the spleen of female MRL mice. (**A**) Cell clusters identified in control- or BaP-treated spleen from female MRL mice, with their individual percentage shown in (**B**). (**C**) Top gene ontology categories enriched in genes downregulated by BaP treatment in female MRL spleen, with example genes shown in (**D**). (**E**) Validation of BaP-downregulated, immune-associated genes by qPCR. (**F**) Immunostaining of CD3 in female or male control- or BaP-treated spleen (4×), with quantification in (**G**). F, female. M, male. Control, vehicle-treated. BaP, BaP-treated. * *p* < 0.05, Student’s *t*-test.

Strikingly, BaP exposure reduced the number of cell types in the spleen of female MRL mice identified by spatial transcriptomic analysis, with six cell-type clusters detected in the control-treated mice and only three detected in the BaP-treated mice (Figure 5A, Supplementary Figure S5). The shared clusters include cluster 1 in the control- and BaP-treated conditions, marked by the megakaryocyte marker *Pf4* and the hemoglobin gene *Hbb-bs* [50] (Figure 5B, Supplementary Figures S5 and S6A). This cluster of cells makes up 28% of all splenic cells in the control, compared to 56% in the BaP-treated condition (Figure 5B). Its two-fold expansion in the BaP-treated spleen is consistent with the histone genes *Hist2h2ac*, *Hist1h3c*, *Hist1h4f*, *Hist2h2bb*, and *Hist1h3d*, which are the top genes enriched in the treated cluster, above *Hbb-bs* and *Pf4*, which suggests extensive cell replication upon BaP exposure (Figure 5B, Supplementary Figure S5). This result is in line with the expansion of red pulp in the BaP-treated spleen by H&E staining (Figure 3A).

Also shared between the two conditions is the cluster of cells expressing the B cell markers *Cd19* or *Cd79a* [51,52,53], making up 21% of all cells in the control-treated (cluster 2) and 21% of all cells in the BaP-treated (cluster 3) spleen, respectively (Figure 5B, Supplementary Figure S6B,C). The third shared cluster lacks significant markers (cluster 6 in control-treated and cluster 2 in BaP-treated).

Three clusters of cells were lost upon BaP treatment, including cluster 2 cells that carry the Th1 cell-specific transcription factor *Txk* [54,55], the T cell receptor genes *Cd3d* and *Cd3e* [56] as well as *Gpr183* and *Cxcr5*, which are known to express in T cells [57,58], cluster 4 cells, which share a gene signature with cluster 2 cells and are high in *Ccr7* or *Ccl5*, and cluster 5 cells, which are *Cd19*/*Cd79a*-negative and express inflammatory, macrophage, and dendritic cell markers including *Cd72*, *Aif1*, and *Cxcl16* [59,60,61] (Figure 5B, Supplementary Figures S5 and S6D).

Differential gene expression analysis identified 230 genes upregulated and 202 genes downregulated upon BaP treatment (FDR < 0.05, Supplementary Figure S7A). Gene ontology analysis showed that BaP-upregulated genes were enriched in functional categories including erythrocyte development and heme biosynthesis, which is consistent with the expansion of red pulp by histological and cluster 1 cells identified by spatial transcriptomic analyses (Supplementary Figure S7B).

In addition, BaP-downregulated genes were enriched in functional categories including adaptive immune response, with a near-complete depletion of *Cd3e* and *Ccl5* expression in the treated spleen (Figure 5C,D, Supplementary Figure S7C). These data are consistent with the loss of white pulp, including cells bearing cluster 3 and 4 signatures, observed from histological and spatial transcriptomic analyses. Similar to the female-biased disruption in spleen cell organization from H&E staining, the loss of expression of immune response genes including *Cd3d*, *Cd3e*, *Lck*, and *Ccl5* was only significant in females by qPCR analysis (Figure 5E). The female-specific loss of CD3-positive cells was further confirmed by immunofluorescence staining (Figure 5F,G). Distinct from the T cell compartment, our cell clustering data suggested that the compartment of CD19^+^ cells was maintained upon BaP treatment, which was also shown by immunofluorescence staining (Figure 5B, Supplementary Figures S6B and S8).

Overall, the spatial transcriptomic analysis of spleens from MRL mice showed that BaP treatment reduced cell-type diversity in females, with T cells being the primary target. While the total number of CD19^+^ cells was maintained, this result does not exclude the possibility that the functions of splenic B cells are impacted by BaP treatment.

### 2.4. BaP Treatment Reduced Renal Cell-Type Diversity and Caused Male-Biased C3 Deposition in the Kidney of MRL Mice

**Figure 6:**
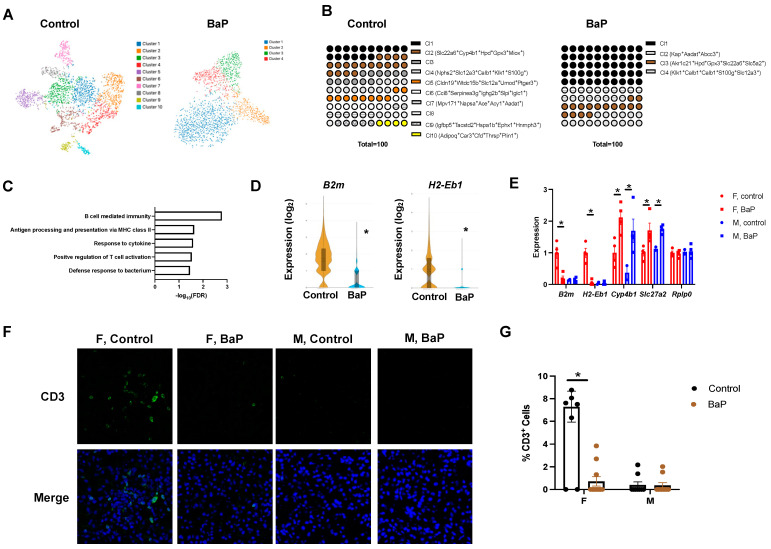
BaP treatment reduced cell-type diversity in the kidney of female MRL mice. (**A**) Cell clusters identified in the control- or BaP-treated kidney from female MRL mice, with their individual percentage shown in (**B**). (**C**) Top gene ontology categories enriched in genes downregulated by BaP treatment in the female MRL kidney, with example genes shown in (**D**). (**E**) Validation of BaP-regulated genes by qPCR. (**F**) Immunostaining of CD3 in female or male control- or BaP-treated kidney (10×), with quantification in (**G**). F, female. M, male. Control, vehicle-treated. BaP, BaP-treated. * *p* < 0.05, Student’s *t*-test.

As observed in the spleen, the exposure to BaP drastically reduced the number of cell types in the kidneys of female MRL mice, with ten cell-type clusters detected in the control-treated and four detected in the BaP-treated condition from the spatial transcriptomic study (Figure 6A,B, Supplementary Figure S9). The shared clusters include the control-treated cluster 2 (making up 18% of all cells in the control kidney) and BaP-treated cluster 3 (making up 16% of all cells in the treated kidney) cells that are positive for the proximal renal tubule organic anion transporter *Slc22a6* [62], control cluster 4 (12%) and BaP cluster 4 (16%) cells that both express the glomerular permeability regulator *Nphs2* [63], and control cluster 7 (8%) and BaP cluster 2 (18%) cells that share the proximal tubule aminoadipate aminotransferase *Aadat* [64] (Figure 6B, Supplementary Figure S9).

Clusters lost in the BaP-treated kidney include cluster 5 cells, which are high in the distal tubule-expressed GPI-anchored glycoprotein *Umod* and prostaglandin E receptor *Ptger3* [65,66], cluster 9 cells that are positive for the insulin-like growth factor binding protein *Igfbp5*, which is known to affect diabetic kidney disease progression [67], and cluster 10 cells which express the proximal tubule carbonic anhydrase *Car3* [68]. In addition, there was a loss of cluster 6 cells which were *Ccl8*^+^ *Iglc1*^+^ and co-expressed *Cd3e*/*Cd3d* or *Cd79a*/*Cd79b* (Figure 6B, Supplementary Figures S9 and S10), indicating an immunosuppressed state that was also supported by H&E staining.

Consistently, the gene ontology analysis of differentially expressed genes demonstrated that BaP treatment downregulated genes involved in B cell-mediated immunity, antigen processing and presentation via MHC class II, the response to cytokine, the positive regulation of T cell activation, and the defense response to bacteria (Figure 6C,D, Supplementary Figure S11A,B). BaP-upregulated genes were enriched in the organic acid metabolic process and benzene-containing compound metabolism, indicating that the kidney is one primary target of BaP toxicity (Supplementary Figure S11C). The BaP-mediated suppression of immune genes was confirmed in female mice, whereas the upregulation of BaP-metabolism genes was observed in both sexes (Figure 6E). Furthermore, the immunostaining of CD3 supported the reduction in T cell numbers in the inflamed female MRL kidneys (Figure 6F,G).

**Figure 7:**
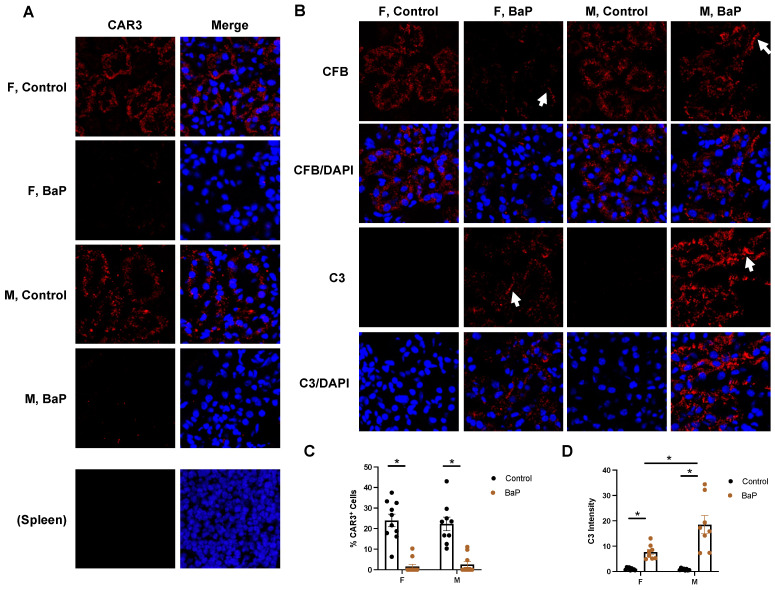
BaP-caused loss in renal tubule cell subtypes associated with an increase in C3 deposition. (**A**) Immunostaining of CAR3 in female or male control- or BaP-treated kidney, with staining in the spleen as a negative control and with quantification in (**C**) (20×). (**B**) Immunostaining of CFB and C3 in female or male control- or BaP-treated kidney, with arrows pointing to fibrils formed and quantification in (**D**) (20×). F, female. M, male. Control, vehicle-treated. BaP, BaP-treated. *, *p* < 0.05, Student’s *t*-test.

Our cell clustering data indicated the loss of a specific proximal tubule cell subtype (*Car3*^+^ cells) despite the presence of other proximal tubule cells (*Slc22a6^+^* and *Aadat^+^*) (Figure 6B, Supplementary Figure S12A). The BaP treatment of human cultured kidney cells (HEK293T) in vitro downregulated a subset of the BaP targets in MRL kidneys, including *PTGER3* and *CAR3*, suggesting that pro-autoimmune processes in vivo facilitate the full activation of BaP targets (Supplementary Figure S12B). Immunofluorescence staining confirmed the BaP-disrupted expression of CAR3 in both female and male MRL mice (Figure 7A,C). To understand whether renal tubule changes associate with lupus disease progression, we performed immunostaining of CFB and C3. Intriguingly, while CFB was diffusely stained in the renal tubule cells under the control condition, BaP exposure triggered the formation of CFB^+^ fibrils, which was more obvious in males (Figure 7B). In addition, BaP treatment caused the deposition of C3, also in the form of fibrils, and the deposition was again most dramatically seen in males (Figure 7B,D). Because C3 accumulation is a hallmark of lupus nephritis, our results may provide a molecular explanation for the male sex being a risk factor for renal failure in lupus patients [36,37].

## 3. Discussion

With genetics only accounting for approximately thirty percent of all autoimmune diseases, it is important to understand the impact of environmental factors on disease pathogenesis [2,3,4]. In this study, we subjected the autoimmune-prone MRL mice to BaP exposure before lupus onset and found that the kidneys were a major organ directly affected by the metabolism of benzene-containing compounds, with the increased expression of BaP-target genes including *Cyp4b1* and *Hao2*. Spatial transcriptomic data demonstrated organ-specific effects of BaP, including the drastic reduction in the diversity of immune and renal cells in the spleen and kidney. The loss of renal tubule cell subtypes was concomitant with the formation of CBP-fibrils as well as significant C3 deposit in a male-biased manner. Together with the male-biased stimulation of pro-tumorigenic genes by BaP in the lung, these data may help explain the increased likelihood of end organ damage in males with lupus.

The MRL mouse model features a late lupus onset, and the BaP treatment procedure was completed before its manifestation of lupus phenotypes such as a significant elevation of a-dsDNA and proteinuria levels. The observation that BaP exposure caused cellular and molecular changes in end organs before disease manifestation suggests that it is important to reduce exposure to BaP in lupus-prone individuals, even in early life. For example, it may be possible to reduce one’s risk of developing autoimmune diseases by limiting smoking and avoiding smoke and combustion fumes. Relatedly, because detrimental cellular and molecular alterations can occur before symptoms and signs of overt disease develop, there is a need to establish novel disease markers for the improved prevention and early detection of autoimmune diseases. Currently, the anti-dsDNA antibody is a commonly used biomarker for SLE based on its widespread presence in patients and association with the disease [24,25]. However, there are subgroups of SLE patients without pathological levels of these autoantibodies, and these antibodies can also be detected in patients with autoimmune hepatitis [30,31,69]. Consistently, our data show that in the female MRL mice, substantial immune infiltrates can occur in multiple organs without a significant elevation in a-dsDNA levels. This result confirms the need to develop more sensitive molecular markers that reflect early organ changes in SLE before pathological levels of a-dsDNA antibodies are detected so that preventative and disease monitoring strategies can be employed in a timely manner.

Similar to previous findings [12,13,14], our study shows an immunosuppressive effect of BaP on autoimmune-prone mice. Further, we identify splenic T cells as a primary target of BaP, with a near-complete destruction upon BaP exposure. While the number of CD19^+^/CD79^+^ B cells in the spleen is maintained, their development and function are likely to ultimately be disturbed, especially with the loss of CXCR5^+^ T_FH_ cells [57]. Therefore, although there is a reduction in the number of inflammatory infiltrates in the lungs and kidneys of MRL mice upon BaP treatment, BaP disrupts the immune system and is by no means beneficial for lupus-prone individuals. Indeed, people with lupus are more likely to experience infection and infection-related complications [70]. The molecular basis for these lupus-associated infections is difficult to establish from human subject studies, often because of the potential complication of immune-weakening medication used on the patients. The MRL mouse model, on the other hand, allows us to probe into the mechanisms underlying immune system perturbation in SLE without the potential complication of medications. Using this model, our study provides a unique perspective on the cellular and molecular targets through which BaP exerts its immunosuppressive effects on lupus-prone subjects and raises the importance of limiting exposure to BaP in protecting lupus patients from severe infections.

One key feature of SLE is its strong female predominance, which is recapitulated by the MRL model [24,25,32,33,34]. While SLE is more common in women, the disease is more severe in male patients, with an increased risk for disease progression to renal failure and lung cancer, especially among smokers [32,35,36,37]. Intriguingly, our study shows that BaP exposure upregulates the expression of angiogenesis genes including *Efnb2* and *Id1* in the lungs and increases the deposition of C3 in the kidneys of MRL mice in a male-biased manner, suggesting that these pathways are key mediators of BaP toxicity. While the MRL mouse allows us to study the effect of BaP on autoimmunity without the potential complication of medications, future projects are needed to ultimately translate these findings into the clinic. For example, it is tempting to test the hypothesis that smoking increases the risk for SLE disease progression through vascular remodeling and complement dysregulation. In addition, the observation that the stimulation of genes including *Id1* by BaP is conserved in human culture cells sets the stage for future attempts to help reduce the risk of smoking-associated lung cancer progression by targeting *Id1* in SLE patients. In summary, our results provide a molecular explanation for the exacerbating effect of BaP exposure on autoimmune disease progression. They reveal sex-biased molecular pathways which may help explain the increased likelihood of end organ damage in males with lupus and prompt future studies to develop strategies for disrupting BaP-stimulated angiogenesis and complementing activation in SLE prevention and treatment.

## 4. Materials and Methods

### 4.1. Animals

MRL mice were purchased from the Jackson Laboratory and subjected to BaP treatment over the course of 8 weeks, starting at 8 weeks of age, following established procedures [13,14,38,39]. Mouse body weight was monitored biweekly, and white blood cell count was performed at week 4 and week 8 of treatment. Plasma was collected for a-dsDNA level by ELISA, and urine was collected for proteinuria analysis at 6 weeks. At the end of the treatment, terminal blood collection was performed, and tissues were snap-frozen for RNA analysis, OCT-embedded for immunofluorescence analysis, and formalin-fixed and embedded for H&E staining and spatial transcriptomic analysis. All animal experiments were conducted with IACUC approval.

### 4.2. ELISA

a-dsDNA ELISA was performed using the mouse anti-dsDNA total Ig’s ELISA quantitative kit (AlphaDiagnostics, cat #5110), following the manufacturer’s instructions. The ELISA assay is based on the binding of mouse anti-dsDNA IgG, IgA, and IgM in samples to dsDNA immobilized on the microwells, and total anti-dsDNA antibody is detected by the anti-mouse IgG + IgA + IgM (H + L)-specific antibody conjugated to the horseradish peroxidase (HRP) enzyme. After adding the chromogenic substrate TMB, the color developed by the enzymatic reaction of HRP on the substrate is directly proportional to the amount of anti-dsDNA Ig present in the sample and calculated relative to anti-dsDNA calibrators.

### 4.3. CLIFT

The *Crithidia luciliae* immunofluorescence test was performed using a substrate in the Kallestad *Crithidia luciliae* substrate kit (Biorad #31069). Anti-dsDNA binding was detected using the anti-mouse IgG + IgA + IgM (H + L)-specific antibody conjugated to FITC and scored relative to the serial dilution of anti-dsDNA-positive calibrators.

### 4.4. Spatial Transcriptomics

Mouse tissues were embedded and sectioned following the Visium tissue preparation guidelines. Tissues were subjected to staining and imaging, permeabilization, reverse transcription, and cDNA amplification. Further, sequencing libraries were prepared by fragmentation, indexing, cleanup, and quality control analysis, following the 10× genomics Visium library construction protocols. Libraries were sequenced on the NovaSeq sequencer, and bioinformatic analysis was performed using the Space Ranger pipeline. Data were visualized in Loupe Browser.

### 4.5. Quantitative Polymerase Chain Reaction (qPCR)

Total RNA was extracted with the Purelink RNA Mini Kit and reverse-transcribed using the SuperScript IV First-Strand Synthesis System, following the manufacturer’s instructions. qPCR reactions were carried out using SYBR Green PCR Master Mix on QuantStudio 7 Flex Real-Time PCR.

### 4.6. Immunofluorescence

OCT-embedded tissues were fixed in 4% paraformaldehyde in PBS, blocked in PBST—1% BSA for 30 min, incubated with primary antibodies overnight, washed 3 × 10 min in PBST, incubated with AlexaFluor conjugated secondary antibodies, following the manufacturer’s recommendations, washed 3 × 10 min in PBS, and then stained with Hoescht. Images were taken using a Zeiss Confocal microscope using appropriate objectives.

### 4.7. Statistical Analysis

Unless otherwise indicated, student’s *t*-test (two-sample, unequal variances) was used to compare experimental (knockdown or inhibition) versus control groups [71,72,73]. The choice of statistical test was based on textbook knowledge and up-to-date literature [72,73,74,75]. Values in the bar graphs are shown as the mean ± s.e.m, with *p* values less than 0.05 indicated with asterisks.

## Data Availability

Not applicable.

## Supplementary Materials

The following supporting information can be downloaded at: https://www.mdpi.com/article/10.3390/ijms24076163/s1.
