# Supplementary material for: Benzo[a]pyrene Exposure Reduces Cell-Type Diversity and Stimulates Sex-Biased Damage Pathways in End Organs of Lupus-Prone Mice
Source: Int J Mol Sci. 2023 Mar 24;24(7):6163. doi: 10.3390/ijms24076163 (PMC10093912; doi:10.3390/ijms24076163)
Supplement: Supplementary file 1 [file ijms-24-06163-s001.zip › ijms-2269854-supplementary.pdf]

Supp Fig S1

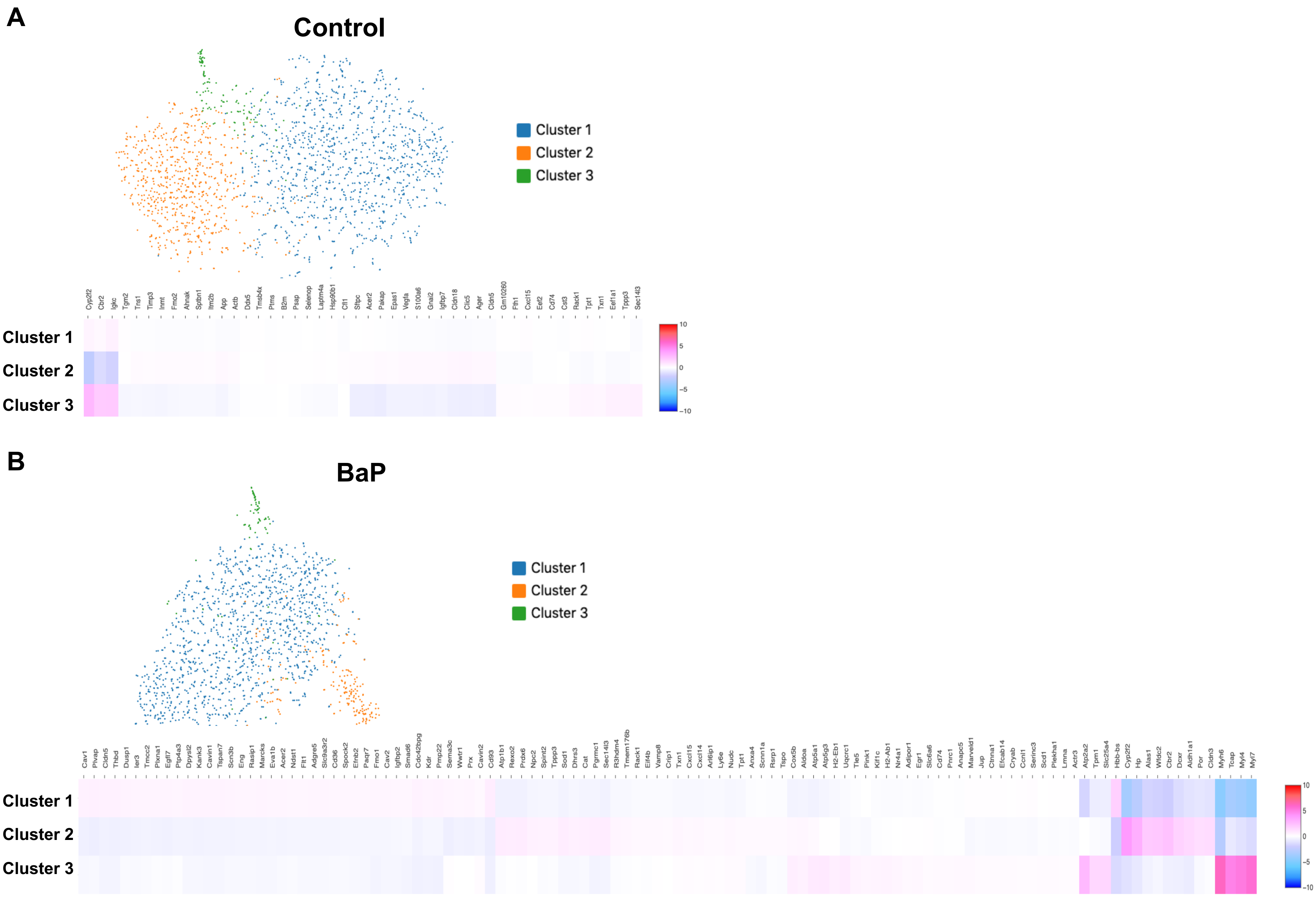

A
Control
Cluster 1
Cluster 2
Cluster 3
Cyp2f2 Cbr2 Igkc Tgm2 Tns1 Timp3 Inmt Fmo2 Ahnak Sptbn1 Itm2b App Actb Ddx5 Tmsb4x Ptms B2m Psap Selenop Laptm4a Hsp90b1 Cfl1 Sftpc Acer2 Pakap Epas1 Vegfa S100a6 Gnai2 Igfbp7 Cldn18 Clic5 Ager Cldn5 Gm10260 Fth1 Cxcl15 Eef2 Cd74 Cst3 Rack1 Tpt1 Txn1 Eef1a1 Tppp3 Sec14l3
10
5
0
−5
−10
B
BaP
Cav1 Plvap Cldn5 Thbd Dusp1 Ier3 Tmcc2 Plxna1 Egfl7 Ptp4a3 Dpysl2 Kank3 Cavin1 Tspan7 Scn3b Eng Rasip1 Marcks Eva1b Acer2 Ndst1 Flt1 Adgre5 Slc9a3r2 Cd36 Spock2 Efnb2 Paqr7 Fmo1 Cav2 Igfbp2 Smad6 Cdc42bpg Kdr Pmp22 Sema3c Wwtr1 Prx Cavin2 Cd93 Atp1b1 Rexo2 Prdx6 Npc2 Spint2 Tppp3 Sod1 Dhrs3 Cat Pgrmc1 Sec14l3 R3hdm4 Tmem176b Rack1 Eif4b Vamp8 Crip1 Txn1 Cxcl15 Cxcl14 Arl6ip1 Ly6e Nudc Tpt1 Anxa4 Scnn1a Rsrp1 Tspo Cox5b Aldoa Atp5a1 Atp5g3 H2-Eb1 Uqcrc1 Tle5 Pink1 Kif1c H2-Ab1 Nr4a1 Adipor1 Egr1 Slc6a6 Cd74 Pnrc1 Anapc5 Marveld1 Jup Ctnna1 Efcab14 Cryab Ccnl1 Serinc3 Scd1 Plekha1 Lmna Actr3 Atp2a2 Tpm1 Slc25a4 Hbb-bs Cyp2f2 Hp Alas1 Wfdc2 Cbr2 Dcxr Aldh1a1 Por Cldn3 Myh6 Tcap Myl4 Myl7

Supplementary Figure S1. Cell clustering analysis of the MRL lung upon BaP treatment. Cell clusters identified in control (A) or BaP (B)-treated female MRL lungs. In each panel, t-SNE cell projection is shown on the top, and signature genes of each cluster are shown below with their overrepresentation (red) or underrepresentation (blue) represented by the heatmap.

Supp Fig S2

A

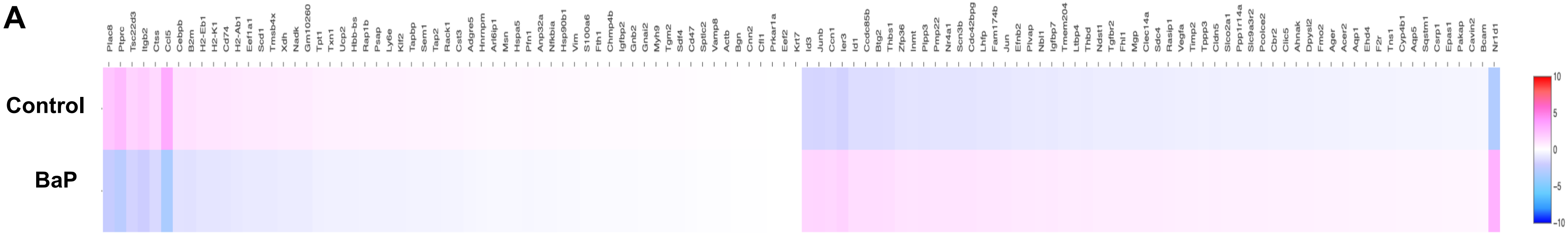

B

GO Biological Process	P-Value	FDR	Gene
Antigen processing and presentation	1.00E-10	1.46E-07	B2m, Cd74, Ctss, H2-Ab1, H2-Eb1, H2-K1
Defense response	2.00E-10	4.97E-07	B2m, Ccl5, Cd74, Cebpb, H2-Ab1, H2-Eb1, H2-K1, Itgb2, Plac8, Ptprc, Scd1
Regulation of developmental process	2.85E-08	4.44E-05	B2m, Ccl5, Cd74, Cebpb, Eef1a1, H2-K1, Itgb2, Pac8, Ptprc, Tmsb4x, Tsc22d3, Xdh
Regulation of leukocyte cell-cell adhesion	3.69E-08	5.03E-05	Ccl5, Cd74, Cebpb, H2-Ab1, Itgb2, Ptprc
Leukocyte activation	5.10E-08	6.11E-05	B2m, Ccl5, Cd74, Cebpb, H2-Ab1, Itgb2, Ptprc

C

GO Biological Process	P-Value	FDR	Gene
Vasculature development	0.00E+00	1.71E-08	Ccn1, clec14a, Efnb2, Id1, Jun, Junb, Ndst1, Nr4a1, Plpp3, Tgfbr2, Thbs1, Tmem204
Cardiovascular system development	0.00E+00	1.71E-08	Ccn1, clec14a, Efnb2, Id1, Jun, Junb, Ndst1, Nr4a1, Plpp3, Tgfbr2, Thbs1, Tmem204
Circulatory system development	0.00E+00	1.67E-07	Ccn1, clec14a, Efnb2, Id1, Id3, Jun, Junb, Ndst1, Nr4a1, Plpp3, Tgfbr2, Thbs1, Tmem204
Developmental process	1.00E-10	1.76E-07	Btg2, Ccc85b, Ccn1, Clec14a, Efnb2, Fhl1, Id1, Id3, Igfbp7, Jun, Junb, Mgp, Nbl1, Ndst1, Nr1d1, Nr4a1, Plpp3, Plvap, Pmp22, Sdc4, Tgfbr2, Thbd, Thbs1, Tmem204, Zfp36
Multicellular organism development	4.00E-10	8.54E-07	Btg2, Ccn1, Clec14a, Efnb2, Fhl1, Id1, Id3, Igfbp7, Jun, Junb, Mgp, Nbl1, Ndst1, Nr1d1, Nr4a1, Plpp3, Pmp22, Sdc4, Tgfbr2, Thbd, Thbs1, Tmem204, Zfp36

Supplementary Figure S2. BaP-regulated genes in the MRL lung. (A) Differentially expressed genes upon BaP treatment of female MRL lungs, with gene name shown on top of the gene expression heatmap. (B) Top biological processes enriched in BaP-downregulated genes in female MRL lungs, with the name of genes in each enriched process as well as the P-Value and FDR for enrichment. (C) Top biological processes enriched included in BaP-upregulated genes in female MRL lungs, with the name of genes in each enriched process as well as the P-Value and FDR for enrichment.

Supp Fig S3

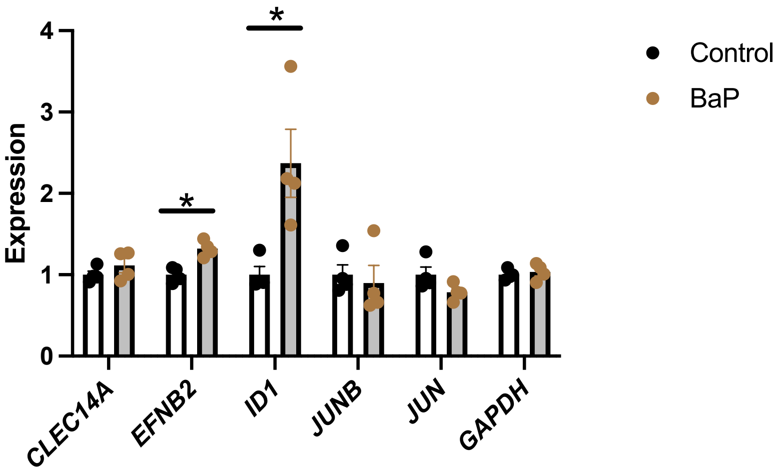

Expression
0
1
2
3
4
*
*
CLEC14A
EFNB2
ID1
JUNB
JUN
GAPDH
Control
BaP

Supplementary Figure S3. Response of pro-angiogenic and pro-tumorigenic genes to BaP in human culture cells. qPCR analysis of the indicated genes upon vehicle (control) or BaP (BaP) treatment in HUVEC culture cells. *, $P < 0.05$, Student's t-test.

Supp Fig S4

A

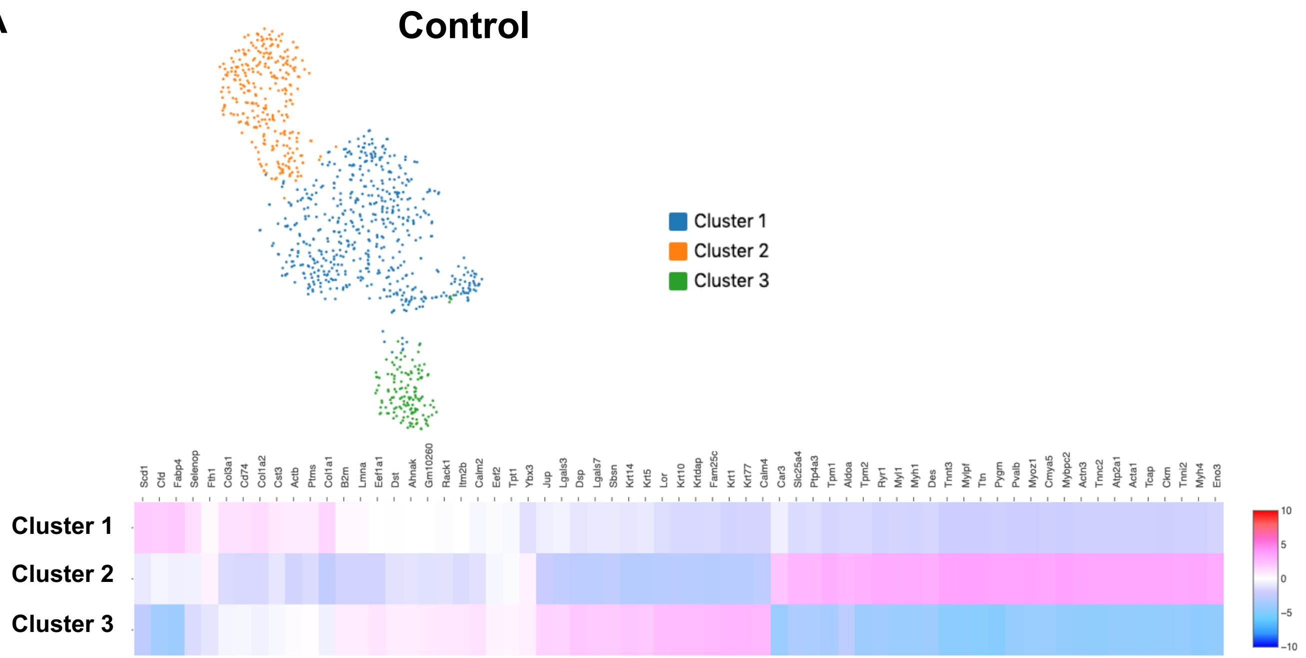

B

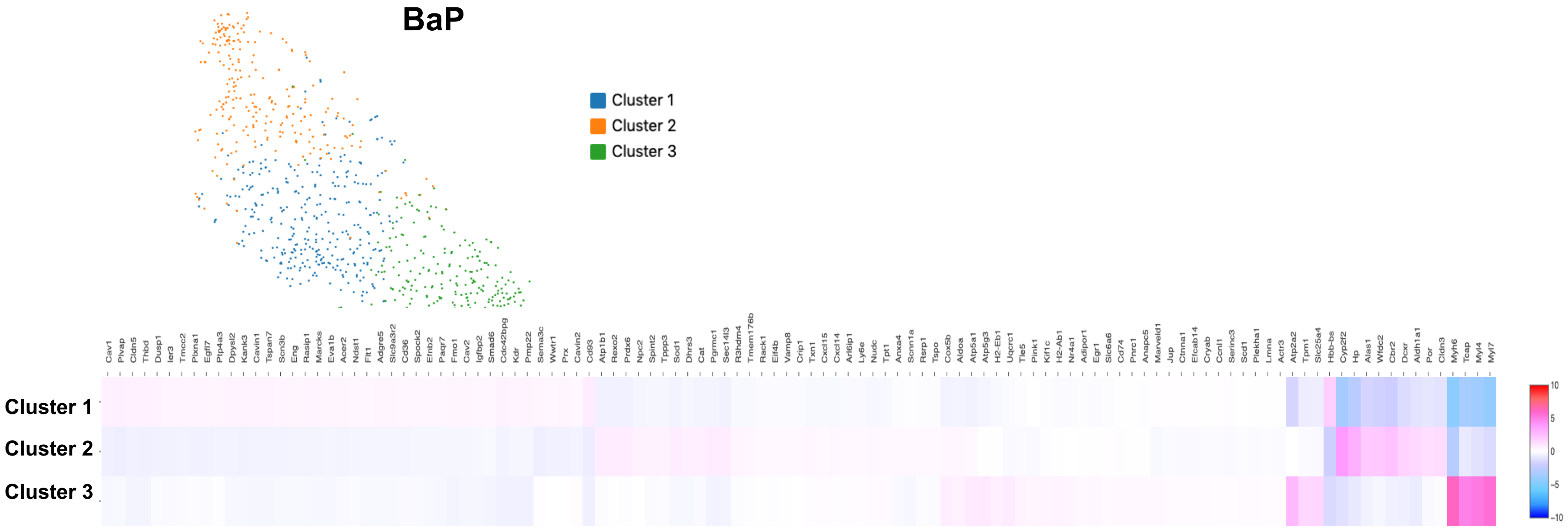

Supplementary Figure S4. Cell clustering analysis of the MRL skin upon BaP treatment. Cell clusters identified in control (A) or BaP (B)-treated female MRL skin. In each panel, t-SNE cell projection is shown on the top, and signature genes of each cluster are shown below with their overrepresentation (red) or underrepresentation (blue) represented by the heatmap.

Supp Fig S5

Control

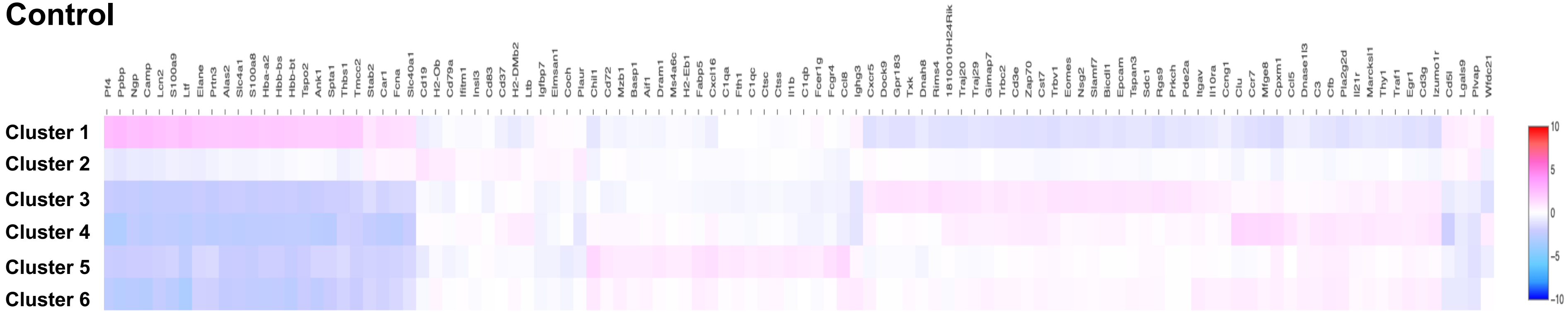

BaP

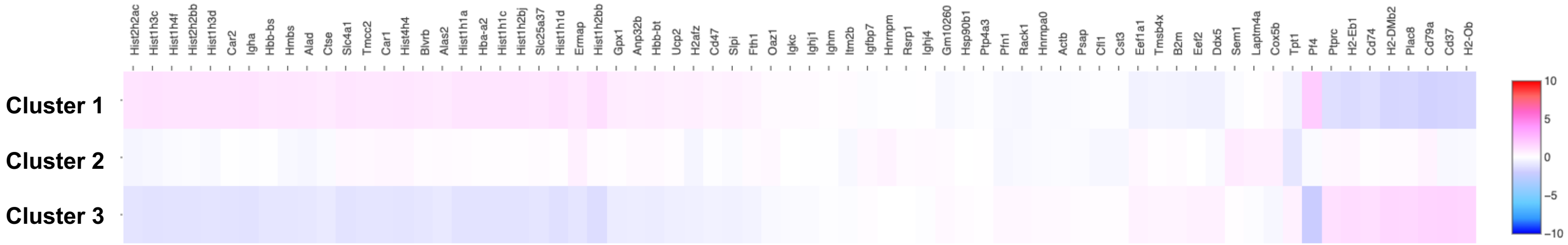

Supplementary Figure S5. Cell clustering analysis of the MRL spleen upon BaP treatment. Cell clusters identified in control (A) or BaP (B)-treated female MRL spleens. In each panel, signature genes of each cluster are shown with their overrepresentation (red) or underrepresentation (blue) represented by the heatmap.

Supp Fig S6

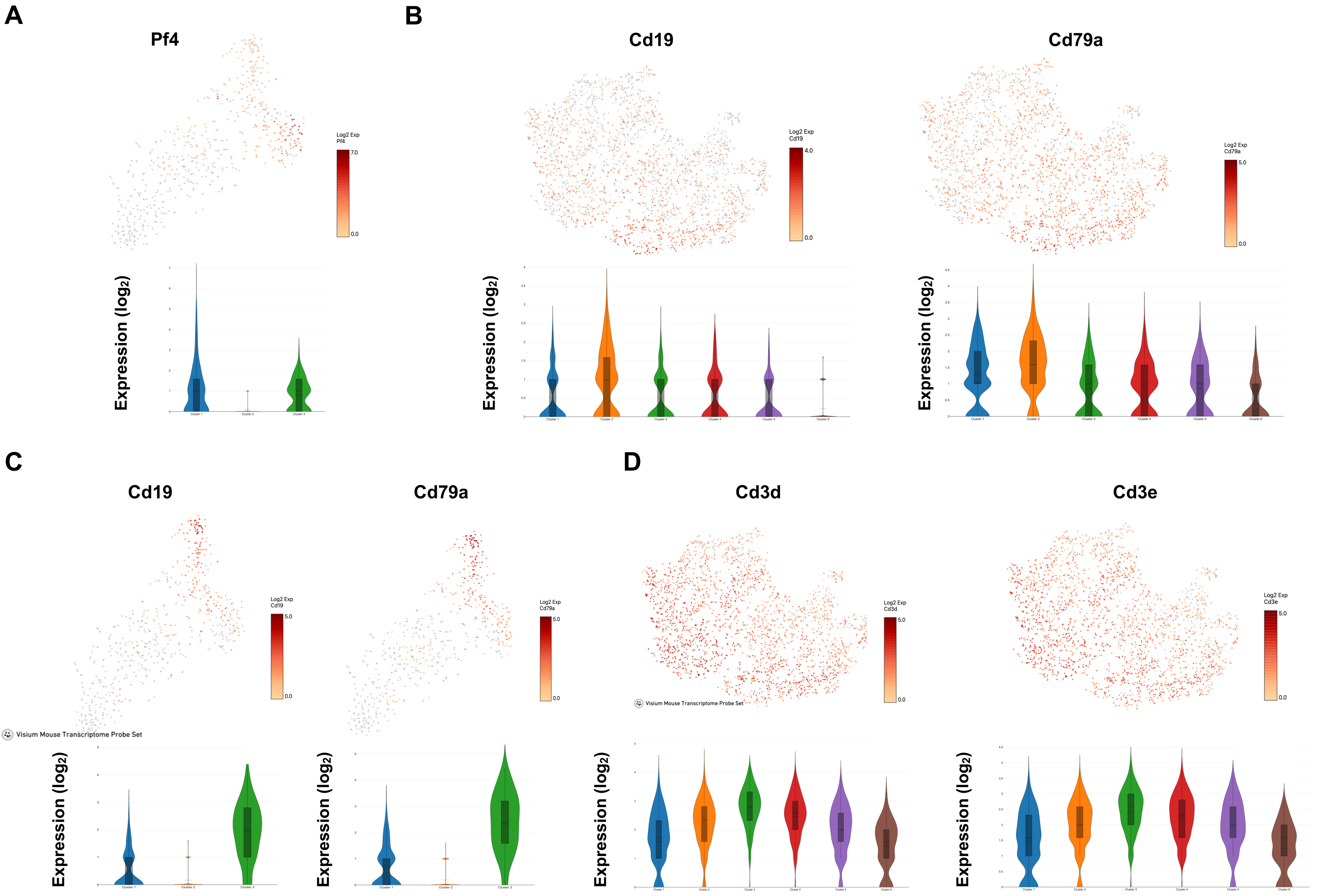
A
Pf4
Log2 Exp
Pf4
7.0
0.0
Expression (log2)
B
Cd19
Log2 Exp
Cd19
4.0
0.0
Cd79a
Log2 Exp
Cd79a
5.0
0.0
C
Cd19
Log2 Exp
Cd19
5.0
0.0
Cd79a
Log2 Exp
Cd79a
5.0
0.0
Visium Mouse Transcriptome Probe Set
D
Cd3d
Log2 Exp
Cd3d
5.0
0.0
Visium Mouse Transcriptome Probe Set
Cd3e
Log2 Exp
Cd3e
5.0
0.0

Supplementary Figure S6. Expression of signature genes in the cell clusters. (A) Expression levels of *Pf4* in each of the clusters of BaP-treated spleen. (B) Expression levels of *Cd19* and *Cd79a* in each of the clusters of the control spleen. (C) Expression levels of *Cd19* and *Cd79a* in each of the clusters of BaP-treated spleen. (D) Expression levels of *Cd3d* and *Cd3e* in each of the clusters of the control spleen.

Supp Fig S7

A

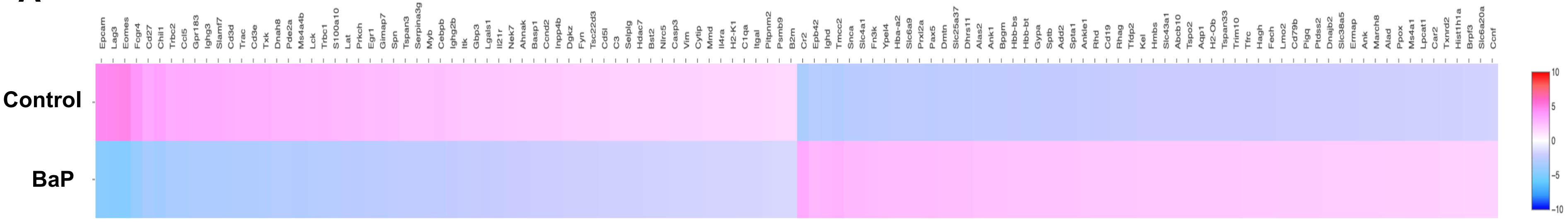

B

GO Biological Process	P-Value	FDR	Gene
Erythrocyte development	1.71E-12	3.00E-09	Epb42, Abcb10, Slc4a1, Dmtn, Ank1, Rhag, Alas2
Heme biosynthetic process	9.12E-08	7.99E-05	Hmbs, Abcb10, Ald, Alas2, Hba-a2, Hbb-bs, Hbb-bt
CO2 and ammonium transport	8.00E-07	7.99E-05	Aqp1, Car2, Rhag
Iron ion homeostasis	1.47E-05	5.65E-03	Epb42, Ank1, Rhag, Alas2
Positive regulation of hemoglobin biosynthetic process	6.82E-05	2.11E-02	Abcb10, Slc25a37, Hba-a2, Hbb-bs, Hbb-bt

C

GO Biological Process	P-Value	FDR	Gene
Adaptive immune response	1.25E-08	2.82E-05	Cd3d, Cd3e, Trbc2, Eomes, Lag3, Ighg3, Slamf6=7
Positive regulation of cell-cell adhesion mediated by integrin	1.31E-05	9.25E-03	Cd3e, Ccl5
Positive regulation of T cell activation	1.46E-05	9.62E-03	Lck, Cd4e, Ccl5, Cd27
Negative regulation of T cell apoptosis	1.51E-04	4.67E-02	Ccl5, Cd27
Neutrophil activation	1.31E-04	4.20E-02	Fcgr4, Ccl5

Supplementary Figure S7. BaP-regulated genes in the MRL spleen. (A) Differentially expressed genes upon BaP treatment of the female MRL spleen, with gene name shown on top of the gene expression heatmap. (B) Top biological processes enriched in BaP-downregulated genes in the female MRL spleen, with the name of genes in each enriched process as well as the P-Value and FDR for enrichment. (C) Top biological processes enriched and genes included in BaP-upregulated genes in the female MRL spleen, with the name of genes in each enriched process as well as the P-Value and FDR for enrichment.

Supp Fig S8

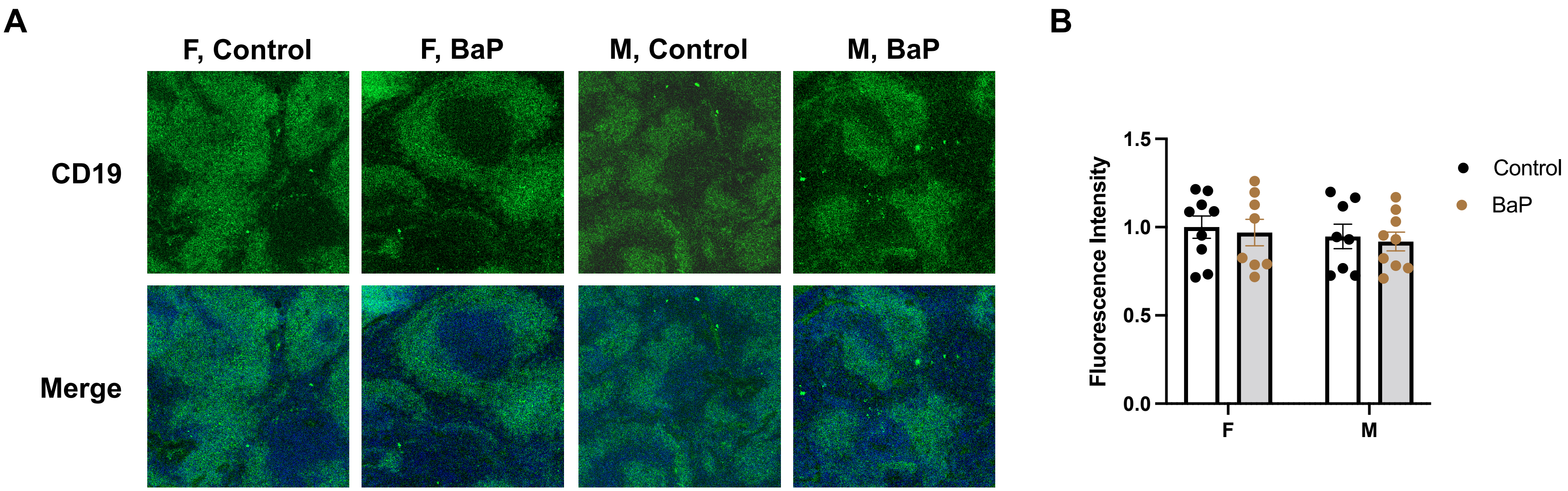
A
F, Control
F, BaP
M, Control
M, BaP
CD19
Merge
B
Fluorescence Intensity
1.5
1.0
0.5
0.0
F
M
Control
BaP

Supplementary Figure S8. Analysis of $CD19^+$ cell in the MRL spleen upon BaP treatment. (A) Immunostaining of CD19 in female or male, control- or BaP-treated spleen, counter-stained by DAPI. Quantification of immunostaining is shown in (B). F, female. M, male. Control, vehicle-treated. BaP, BaP-treated. *, $P < 0.05$, Student's t-test.

Supp Fig S9

Control

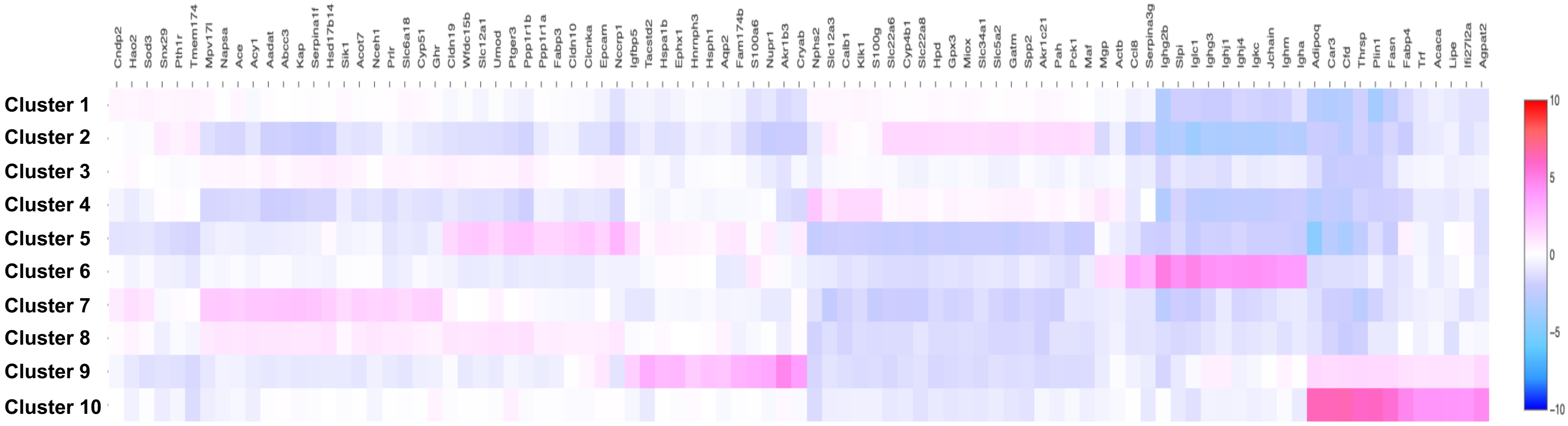

BaP

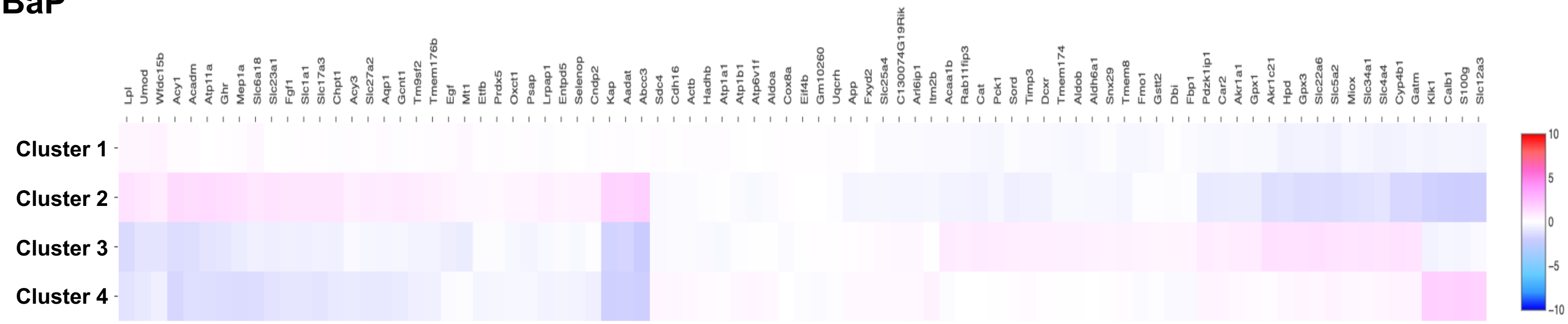

Supplementary Figure S9. Cell clustering analysis of the MRL kidney upon BaP treatment. Cell clusters identified in control (A) or BaP (B)-treated female MRL kidneys. In each panel, signature genes of each cluster are shown with their overrepresentation (red) or underrepresentation (blue) represented by the heatmap.

Supp Fig S10

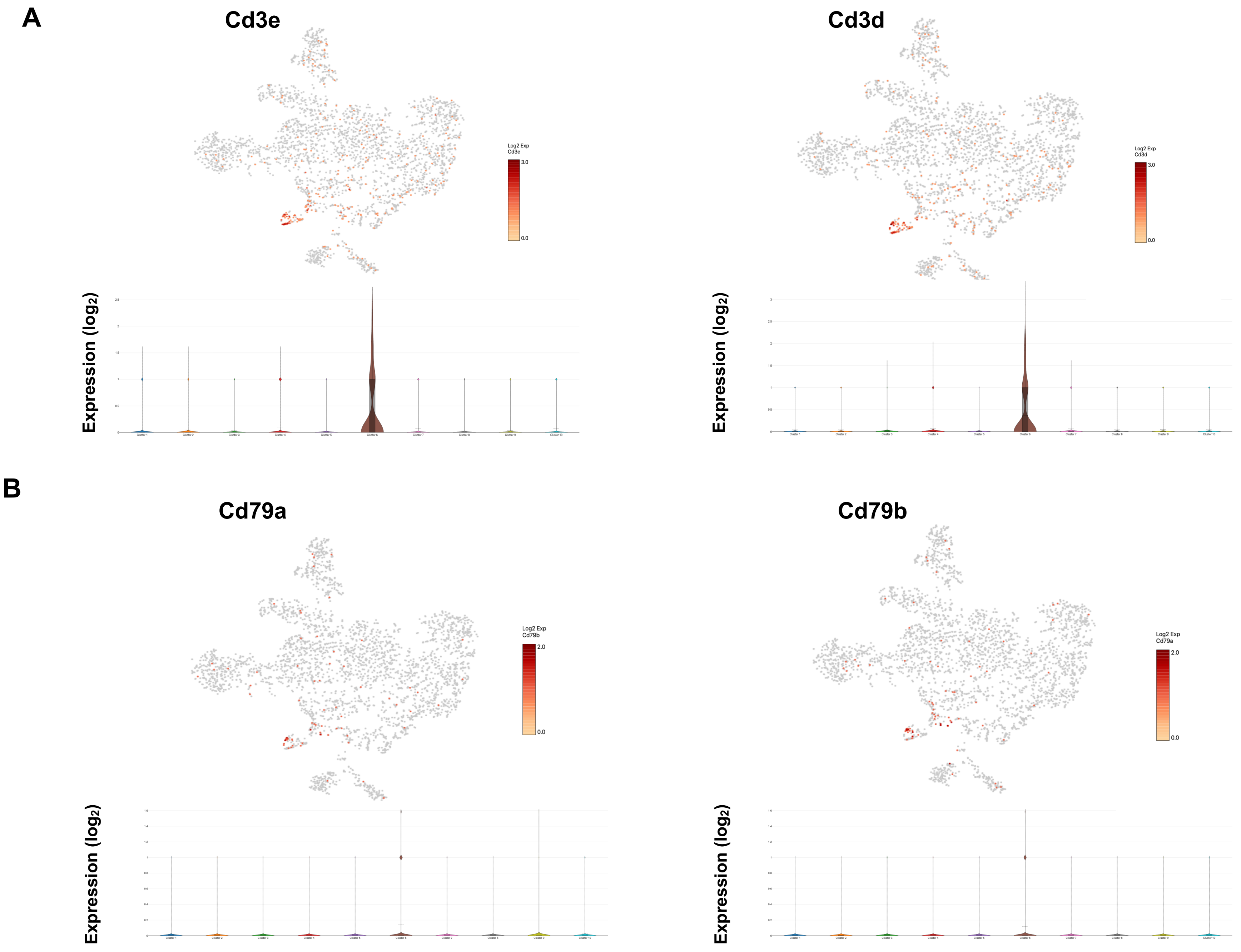

A
Cd3e
Log2 Exp
Cd3e
3.0
0.0
Expression (log2)
Cd3d
Log2 Exp
Cd3d
3.0
0.0
Expression (log2)
B
Cd79a
Log2 Exp
Cd79b
2.0
0.0
Expression (log2)
Cd79b
Log2 Exp
Cd79a
2.0
0.0
Expression (log2)

Supplementary Figure S10. Expression of signature genes in the cell clusters. (A) Expression levels of *Cd3d* and *Cd3e* in each cell cluster of the control kidney. (B) Expression levels of *Cd79a* and *Cd79b* in each cell cluster of the control kidney.

Supp Fig S11

A

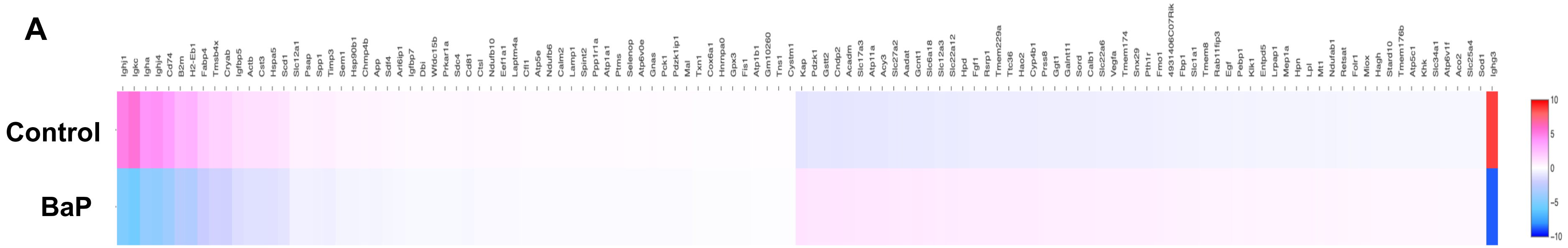

B

GO Biological Process	P-Value	FDR	Gene
B cell mediated immunity	2.09E-07	1.65E-03	B2m, Igha, Cd74, Igkc, H2-Eb1, Ighg3
Antigen processing and presentation via MHC class II	2.65E-06	3.37E-03	B2m, CD74, H2-Eb1
Response to cytokine	5.61E-05	2.60E-02	Cd74, Timp3, Spp1, Fabp4, H2-Eb1, Hspa5
Positive regulation of T cell activation	6.71E-05	2.94E-02	B2m, Cd74, H2-Eb1, Actb
Defense response to bacterium	8.64E-05	3.49E-02	B2m, Igha, Igkc, Scd1, Ighg3

C

GO Biological Process	P-Value	FDR	Gene
Organic acid metabolic process	2.83E-07	4.47E-03	Slc17ac, Hao2, Hpd, Slc27a2, Aadat, Ggt1, Fmo1, Ttc36, Slc22a12, Acadm
Benzene-containing compound metabolic process	8.74E-06	4.59E-02	Cyp4b1, Hao2, Aadat

Supplementary Figure S11. BaP-regulated genes in the MRL kidney. (A) Differentially expressed genes upon BaP treatment of the female MRL kidneys, with gene name shown on top of the gene expression heatmap. (B) Top biological processes enriched in BaP-downregulated genes in the female MRL kidneys, with the name of genes in each enriched process as well as the P-Value and FDR for enrichment. (C) Top biological processes enriched in BaP-upregulated genes in the female MRL kidney, with the name of genes in each enriched process as well as the P-Value and FDR for enrichment.

Supp Fig S12

A

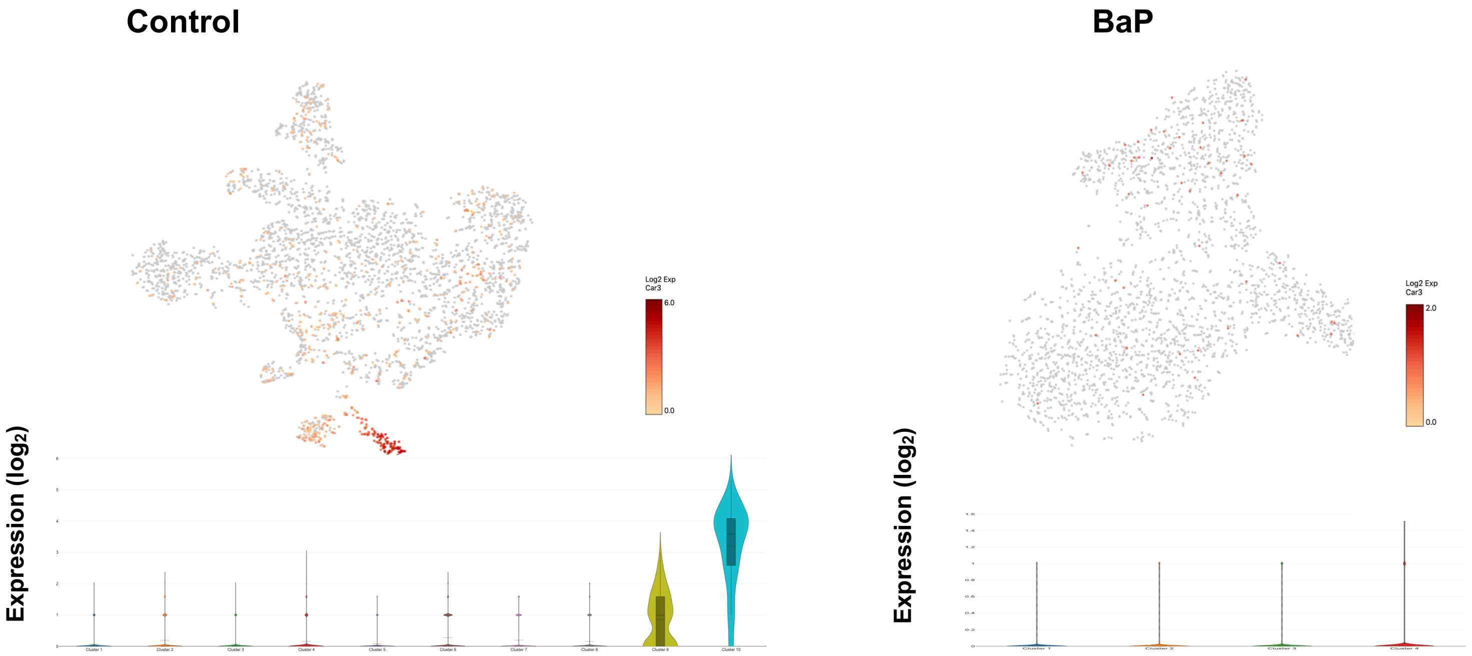

B

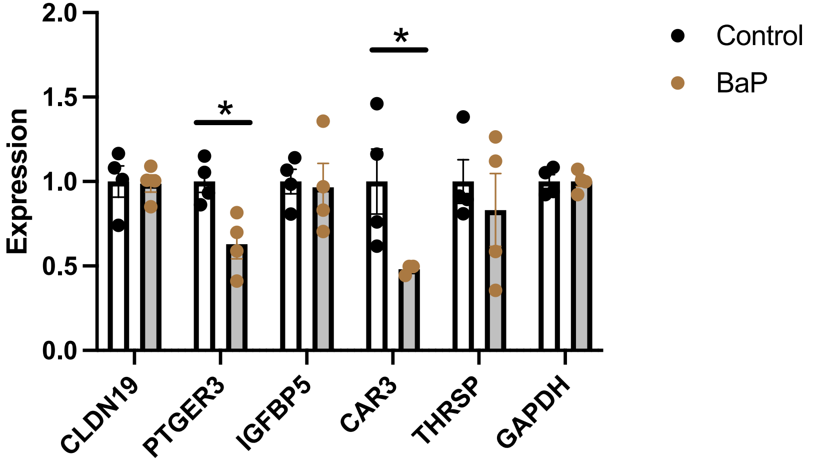

Supplementary Figure S12. Expression of *Car3* in the cell clusters and BaP-regulated genes in human culture cells. (A) Expression levels of *Car3* in each cell cluster of the control (left) or BaP (right)-treated kidney. (B) qPCR analysis of the indicated genes upon vehicle (control) or BaP (BaP) treatment in HEK293 culture cells. *, $P < 0.05$, Student's t-test.
